# Phytoremediation of Heavy Metal-Contaminated Soil by Switchgrass: A Comparative Study Utilizing Different Composts and Coir Fiber on Pollution Remediation, Plant Productivity, and Nutrient Leaching

**DOI:** 10.3390/ijerph16071261

**Published:** 2019-04-09

**Authors:** Paliza Shrestha, Korkmaz Bellitürk, Josef H. Görres

**Affiliations:** 1 Plant and Soil Sciences Department, The University of Vermont, Burlington, VT 05405, USA; jgorres@uvm.edu; 2 Faculty of Agriculture, Department of Soil Science and Plant Nutrition, Tekirdağ Namık Kemal University, Tekirdağ 59030, Turkey; kbelliturk@hotmail.com

**Keywords:** phytoremediation, heavy metals, bioremediation, switchgrass, thermophilic compost, vermicompost, coir

## Abstract

We investigated the effects of organic amendments (thermophilic compost, vermicompost, and coconut coir) on the bioavailability of trace heavy metals of Zn, Cd, Pb, Co, and Ni from heavy metal-spiked soils under laboratory conditions. To test switchgrass (*Panicum virgatum*) as a potential crop for phytoremediation of heavy metal from soil, we investigated whether the addition of organic amendments promoted switchgrass growth, and consequently, uptake of metals. Compost is a valuable soil amendment that supplies nutrients for plant establishment and growth, which is beneficial for phytoremediation. However, excess application of compost can result in nutrient leaching, which has adverse effects on water quality. We tested the nutrient leaching potential of the different organic amendments to identify trade-offs between phytoremediation and water quality. Results showed that the amendments decreased the amount of bioavailable metals in the soils. Organic amendments increased soil pH, electrical conductivity (EC), and soil nutrient status. Switchgrass shoot and root biomass was significantly greater in the amended soils compared to the non-amended control. Amended treatments showed detectable levels of heavy metal uptake in switchgrass shoots, while the control treatment did not produce enough switchgrass biomass to measure uptake. Switchgrass uptake of certain heavy metals, and concentrations of some leachate nutrients significantly differed among the amended treatments. By improving soil properties and plant productivity and reducing heavy metal solubility that can otherwise hamper plant survival, organic amendments can greatly enhance phytoremediation in heavy metal-contaminated soils.

## 1. Introduction

Phytoremediation is a set of ecological strategies that utilizes plants, in situ, to promote the breakdown, immobilization, and removal of pollutants from the environment [1,2,3]. Plants have a more direct effect on contaminant levels via phytoextraction, which concentrates contaminants (e.g., heavy metals) from the environment into plant tissues. Phytoremediation is a cost-effective remediation solution for removing pollutants (mainly heavy metals and organics) from contaminated soils and waters at site level with little disturbance to the landscape [3,4]. It also reduces the cost of alternatively disposing hazardous wastes to a landfill or a storage facility located off-site [3].

Efficient plants for phytoremediation are highly productive, good bioaccumulators with tolerance to high levels of pollution. Switchgrass (*Panicum virgatum*) is known for its high biomass production [5,6] that allows it to remove excess nutrients from sites amended with dairy manure [7]. In the presence of switchgrass, the degradation of herbicide such as atrazine may be accelerated [1]. Other researchers have proposed that switchgrass might extract heavy metals from contaminated soils [6,8]. Switchgrass has also been utilized in bioretention systems for storm runoff treatment in urban and mixed-urban agricultural settings [9,10]. In this paper, we focus on the ability of switchgrass to extract toxic trace heavy metals with and without yield-enhancing organic amendments. Since it is expensive to treat large amounts of heavy metal-polluted soils with conventional techniques of mechanical removal [11] or chemical immobilization [12], the combined in situ approach of using recycled organic waste (compost) and plants is more affordable [13] and may be a promising phytoremediation strategy.

The efficiency of phytoremediation using switchgrass or other plants on contaminated soil can be enhanced through additions of composts and other organic matter sources (e.g., coir) that are locally and cheaply available depending on the region. The proposed mechanism is that plant heavy metal uptake and assimilation increases with biomass. Composts differ both in the feedstock materials and the processes used to create them. There are two common aerobic processes to produce composts. Thermophilic composts encourage thermophilic microorganisms to decompose organic wastes (temperatures reaching 45 to 70 °C) followed by a mesophilic maturation process [13] where organic matter becomes more stable and may resist further decomposition. Vermicomposting relies on earthworms and their gut flora to decompose the organic wastes but is frequently preceded by a thermophilic stage (temperatures between 25 to 40 °C [13,14]) when organic certification is required. This process occurs at mesophilic temperatures and fosters a very different microbial community [15]. In broad strokes, thermophilic composts are mature at C:N ratios between 15–20:1 [16] and have low available nitrogen content. In contrast, vermicompost is mature at CN ratios of 10–15:1 [17] and has high available nutrient contents. However, these benchmarks may differ depending on the feedstocks.

This paper reports on a lab study that explores the efficacy of switchgrass to remove heavy metals from soils amended with composts and coir. Composts contribute to soil quality by improving aeration, moisture-holding capacity, carbon supply, microbial activity, cation exchange capacity, and supplies macro and micronutrients [18,19,20,21,22] in the soil for plant growth. Survival of plant growth on contaminated soils may differ upon the quality and type of compost utilized. Thus, compost may increase plant contaminant uptake by stimulating plant productivity, while compost itself can also directly influence bioremediation [21,23,24,25]. The humic substances in compost can remove heavy metals in dissolved forms from the soil solution [26,27,28] through complexation, sorption, and precipitation [23,25,29], rendering them less mobile, thereby posing less threat to the environment [24,28,30]. However, this may also counteract the ability of a phytoextracting plant to remove the metals.

Coir has also been shown to be a promising bio-adsorbent for remediation of heavy metals. Coir is the fiber that is derived from the inner shell of the coconut, which may be added as a substrate to compost soils to enhance its performance. Previously considered a waste product and, as a result, dumped or incinerated, new uses have been developed over the last decade, including using the coir as a soil amendment for degraded soils [31]. Most results are, however, inferred from laboratory batch sorption experiments using aqueous solutions containing heavy metals [32,33] with concentrations similar to those of wastewaters [34]. Coir is an organic waste product that may be added as a substrate to compost soils to enhance soil and plant performance. Coir is a source of organic matter, and though it contains few nutrients itself, it has high nutrient retention capacity [31,35], and improves the overall quality of the soil, although it alone cannot be a sufficient growing media [36]. Coir is resistant to environmental biodegradation; as a result, the slow breakdown of coir can also release a steady supply of carbon. The proposed mechanism in the case of this research is that coir has a high C:N ratio substrate (ratio of 75 to 186 [31,37]), therefore rendering greater microbial immobilization of metals and nutrients from the soil to enhance phytoremediation benefits.

The main objective of our experiment was to investigate whether promoting plant growth by organic matter additions increases the uptake of heavy metals. Organic additions included thermophilic compost (hereby called compost), vermicompost, and coir in various combinations. We specifically studied the effects of heavy metals on switchgrass productivity and heavy metal uptake potential in soils with and without organic amendments. Switchgrass was chosen because of its high biomass production capacity. To our knowledge, no study has been conducted evaluating phytoremediation of heavy metals by switchgrass in the presence of different organic soil amendments. In addition, we also examined heavy metal bioavailability and nutrient leaching potential of unvegetated soils treated with different organic amendments to examine possible trade-offs between phytoremediation and water quality.

## 2. Materials and Methods

### 2.1. Experimental Design

**Figure 1:**
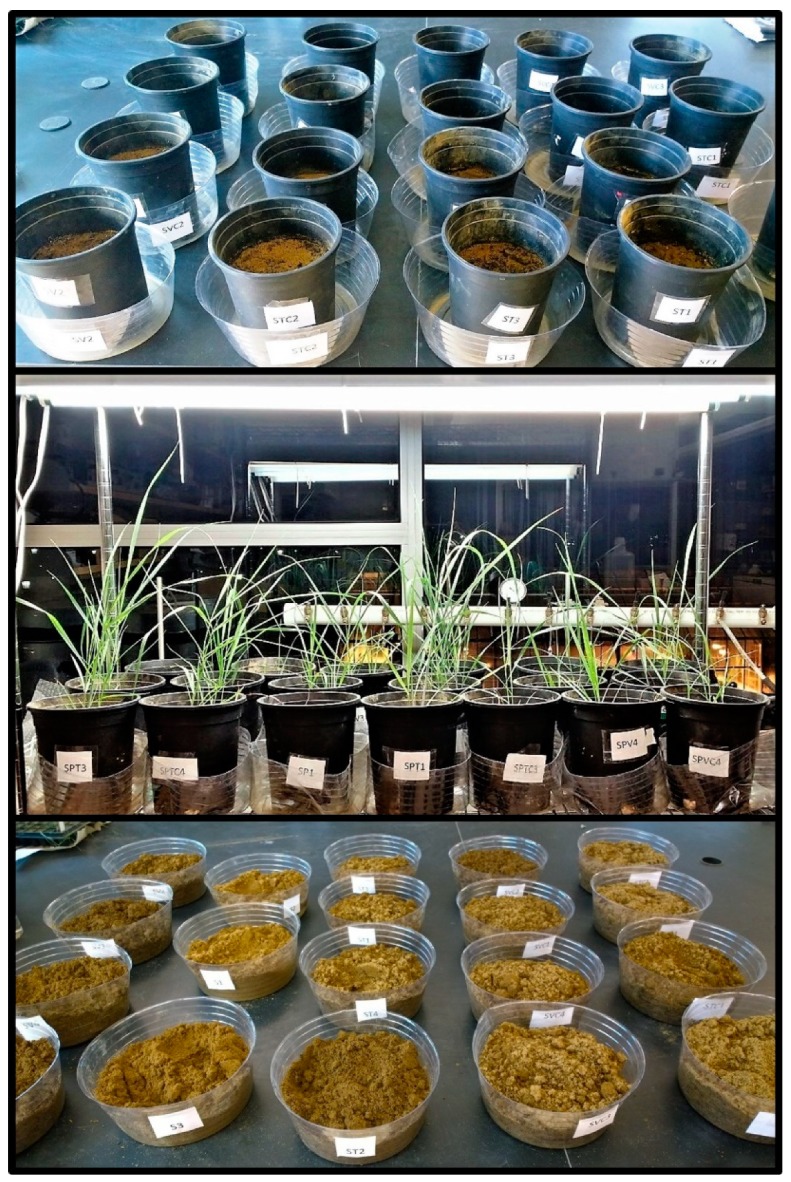
(**Top**): Pots containing contaminated soils amended with the different organic treatments without switchgrass placed over plastic containers used for leachate collection, (**Middle**): Vegetated pots containing switchgrass growing in laboratory under 24-h light conditions, (**Bottom**): Soil that was removed from the pots at the end of the experiment for analysis.

The following laboratory experiment is a complete block design with 10 treatments replicated four times (Table 1; Figure 1) resulting in 40 pot-scale, experimental units. The experiment examines blends of thermophilic compost (T), vermicompost (V), and coir (C) mixed in different combinations (substrate chemical properties outlined in Table 2) with and without switchgrass. The resulting treatments are soil (S), soil + thermophilic compost (ST), soil + thermophilic compost + coir (STC), soil + vermicompost (SV), and soil + vermicompost + coir (SVC) (Table 1). Thermophilic compost was collected from Green Mountain Compost Facility located in Williston, Vermont. Vermicompost was obtained from Worm Power, an organic composting facility located in Avon, New York. Coir was purchased from Gardeners Supply Company located in Burlington, Vermont.

### 2.2. Soil Collection and Pot Culture Preparation

Native soil was collected from a mixed hardwood forest located adjacent to University of Vermont Horticulture Research Center, Burlington, USA. The soil is a very well-drained Windsor (mixed, mesic Typic Udipsamments) series [38] with low organic matter content of 0.7%, suggesting that the soil is low in nutrient availability (Table 2). The fine earth fraction of the soils was obtained using a 2-mm stainless-steel sieve as the standard operation procedure (USDA 2014) to have a relatively homogenous sample free of large unreactive particles (e.g., stones) across all treatments. Any stones (or roots) in the pass fraction were further removed by hand. Sifted soils were left to air dry for over a week. The compost samples were also left to air dry in lab conditions for two weeks. The coir, which was purchased as a brick of dried coconut husk fiber, was soaked in de-ionized water to pull the fibers apart, and then left to air dry for over a month. All the air-dried soil, compost, and coir substrates were homogenized before application to treatment pots. Soil or amended soil was added to pots lined with coffee filters (Mellita brown coffee filters). Amended soil was created by mixing 1.5 kg air-dried soil with either 0.12 kg of air-dried compost or vermicompost, and 0.06 kg of air-dried coir (8% and 4% of dry soil weight, respectively) to make up the recipes in Table 1. In non-amended control soil pots, the soil equivalent of these weights was added so that the resulting weight in all pots was 1.68 kg. To each substrate type, switchgrass was either added or not added (Table 1). Each plant by substrate combination had 4 replicates for a total of 20 pots.

### 2.3. Switchgrass Seed Preparation

Switchgrass seeds were grown in small plugs that were pre-filled with the experimental soil. Fifteen switchgrass seeds were sowed into each plug. A total of 4 mL of solution NPK fertilizer (100, 80, 100 ppm, respectively) was added to the soil at the start. NO_3_^−^ was made from 1000 mg L^−1^ pure NO_3_^−^ stock solution. P and K were made from KH_2_PO_4_ powder by mixing 0.349 g of the compound into 1 L de-ionized water. The plugs were transported to the University of Vermont (UVM) campus greenhouse. They were irrigated daily, kept in 12-h day/night cycle, and temperature was maintained at 21 °C. In the greenhouse, plants were not further fertilized until they germinated. Once germinated, plants were fertilized six times, every Monday and Friday for three weeks, using the facility’s standard NPK fertilizer at 17-4-17 at 150 ppm nitrogen.

### 2.4. Phytoremediation Experiment

The different soil mixes in the 40 pots were spiked with 32 mg of five different heavy metals based on soil dry weight. Individual solutions of 0.672 M Zn, Cd, Pb, Co, and Ni were prepared using respective metal salt compounds: Zinc Chloride (ZnCl_2_), Cadmium Chloride (CdCl_2_), Lead Chloride (PbCl_2_), Cobalt Chloride (CoCl_2_·6H_2_O), and Nickel Chloride (NiCl_2_·6H_2_O), respectively. The total mass of the metals in soil for each treatment after contamination is given in Table 3. The total mass of the metals in soil for each treatment after contamination is given in Table 3 (See Appendix A for data on the metal mass of the original substrates before and after combining them to make the recipes in Table A1).

Four days following heavy metal application, two of the plugs containing the largest seedlings (8 to 10 cm) were transplanted into pots (Figure 1). The pots were brought to equal soil moisture content once before planting of the switchgrass to account for the loss of moisture through evaporation. Each plug contained one or two switchgrass plants at the time of transplanting (only a few seeds had germinated in that time out of the 15 seeds that were originally sowed). All vegetated and unvegetated pots were irrigated with 50 mL de-ionized water twice a week for the first two weeks, and then every other day as the plants grew taller. Any leachate collected in the plastic container beneath the pots was poured back into their respective pots. The pots containing switchgrass were kept under 24 h light in the laboratory with the help of growth lights for approximately 7 weeks, and at temperatures around 25 °C (Figure 1).

### 2.5. Plant-Available or Bioavailable Heavy Metals

At the end of the 54-day incubation period, soils from the unvegetated pots were analyzed for metal bioavailability (defined here as plant-available fraction) using a nonaggressive extractant method. This method was chosen to extract the fraction of heavy metals that is less strongly adsorbed to soil and more mobile and therefore of an interest from an environmental water quality standpoint. In contrast, a substantial fraction of the heavy metals extracted using chemically aggressive reagents may not be bioavailable [39], especially under natural environmental conditions. A 10 g subsample of air-dried soils from the unvegetated pots was taken, combined with 25 mL of 0.01 M CaCl_2_ solution, and the suspension was shaken for 24 h on a mechanical shaker at room temperature [39]. Solution was filtered through Ahlstrom filter paper 642 (particle retention of 2 µm), and filtrate was analyzed in triplicates using the inductively coupled plasma optical emission spectrometry (ICP-OES/AES, Optima 3000DV, Perkin Elmer Corp, Norwalk, CT, USA).

### 2.6. Plant Analysis (Tissue Metal Concentrations and Loads)

From the planted pots, switchgrass plants were harvested, and separated into roots and shoots at the end of the 54-day lab incubation period. The plant samples were washed with de-ionized water, oven dried at 70 °C for at least 5 days, and weighed for dry biomass. Dried plant samples were ground and digested (approximately 0.5 g) with 10 mL of 16 N concentrated nitric acid diluted to 50 mL with deionized water, and the extract was used to determine heavy metal concentrations by ICP-OES as per the USEPA SW846-3051A (USEPA 2007) method. Total mass of metal uptake in each of the pots was estimated as the product of plant metal concentrations and plant biomass.

### 2.7. Soil Analysis

The entire soil content from all pots, including those planted to harvest, were transferred into large plastic containers, and mixed thoroughly. Water content was determined gravimetrically for each experimental unit as the difference between fresh and oven-dry mass (about 10 g were dried for 48 h at 105 °C). pH and electrical conductivity (EC) were also determined using 10 g of fresh soil mixed in 20 ml distilled water using Fisher Scientific Accumet Portable APILO (pH/ORP meter) and Thermo Scientific Orion Star A222 Conductivity meter, respectively. The remaining soils in the plastic container were left to air dry for one week before being analyzed for total metals. Soils were ground using mortar and pestle. The ground soil was screened through 0.5 mm sieve and dried at 60 °C for several hours. Total heavy metal concentrations were analyzed using the ICP after following a microwave-assisted digestion of approximately 0.5 g soil in 16N concentrated nitric acid diluted to 50 ml with deionized water [40].

### 2.8. Leachate Nutrient Analysis (NH_4_^+^, NO_3_^−^, PO_4_^3−^)

A leachate experiment was conducted to measure nutrient leaching potential of the different soil treatments following a short pulse of rain event. 700 mL of de-ionized water was evenly applied to the soil surface of the unvegetated pots (Table 1), which was designed to mimic a short rain event that runs through the soil media (methods modified from Hurley et al. [41]). Leachate water was collected in plastic containers placed under each pot (Figure 1). The leachate samples were filtered using a 0.45-µm nylon mesh filter (Fisher Scientific) and analyzed for available dissolved nutrients (NH_4_^+^, NO_3_^−^, PO_4_^3−^) by flow injection analysis on an automated colorimeter (Lachat Instruments QuickChem8000 AE, Hach Inc., Loveland, CO, USA) using the Cd-reduction method for NO_3_^−^, the salicylate-nitroprusside method for NH_4_^+^, and the ammonium molybdate colorimetric method for PO_4_^3−^ [42].

### 2.9. Statistical Analysis

The effects of soil organic amendments on heavy metal bioavailability, soil properties, switchgrass biomass, and metal uptake were analyzed using the analysis of variance (ANOVA) in JMP Pro 13 (SAS Institute Inc., Cary, NC, USA). A Tukey’s Honestly Significant Difference (HSD, α = 0.05) post hoc test was used to test for significant differences in the treatment means. When necessary, log transformations on the data were carried out to satisfy the assumption of normality and equal variance required by ANOVA.

## 3. Results

### 3.1. Bioavailable Metals

The fraction of bioavailable metal mass for all metal species (Zn, Cd, Pb, Co, and Ni) was significantly higher in the control soil compared to organically amended treatments (Table 4). No significant differences in the mass of bioavailable metals were observed among the amended soil treatments. In the control soil, the percentage of total metal mass that was bioavailable was in the range of 0.33% up to 70%, while only 0.04% to 1.02% of total metals mass were bioavailable in the compost-amended soils (Table 3 and Table 4).

### 3.2. Soil pH and EC

All organic amendments significantly increased soil pH (from slightly acidic at 4.65 in the control to more neutral at 6.43) and EC (μS cm^−1^; from approx. 80 in control to upwards of 290 to 900) in both vegetated and unvegetated pots (Table 5). In the unvegetated pots, no significant difference in pH was observed among the organic treatments, while in the plant treatments, greater pH was observed in the compost treatments relative to the vermicompost treatments. Overall, EC was three times higher in vermicompost treatments compared to compost treatments and the increase in EC in vermicompost treatment was only significant in vegetated treatments (Table 5).

### 3.3. Switchgrass Productivity

**Figure 2:**
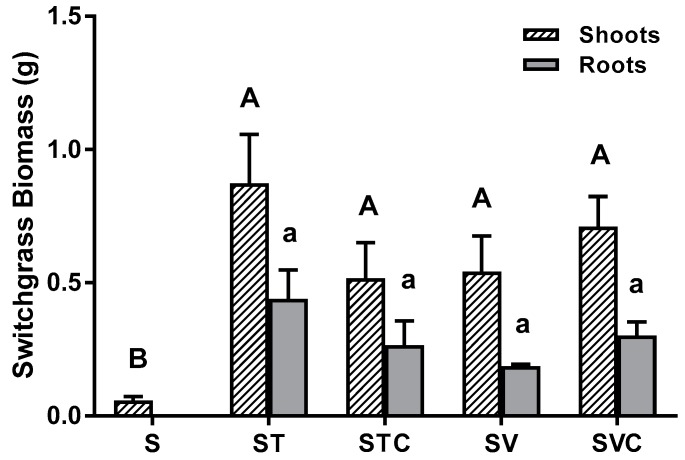
Mean ± 1 S.E. switchgrass shoot and root biomass (g) from control soil (S), and soil amended with thermophilic (T) and vermicompost (V) with and without coir (C). Varying uppercase and lowercase letters indicate significant differences in shoot and root biomass, respectively, across treatments.

All organic amendments improved switchgrass productivity, both aboveground and belowground, over the study duration (Figure 2). Switchgrass shoot and root biomass was significantly greater in the organically amended relative to control treatments, but no differences were observed among the different organic amendments. No harvestable/quantifiable switchgrass roots were recovered from the control treatment, while significantly greater shoot biomass (11 times) was measured from the amended treatments relative to the control treatment (0.661 ± 0.29 g versus 0.058 respectively; Figure 2) Overall, the shoot biomass exceeded root biomass in all the vegetated treatments (Figure 2).

### 3.4. Total Metal Mass in Switchgrass and Soils

**Figure 3:**
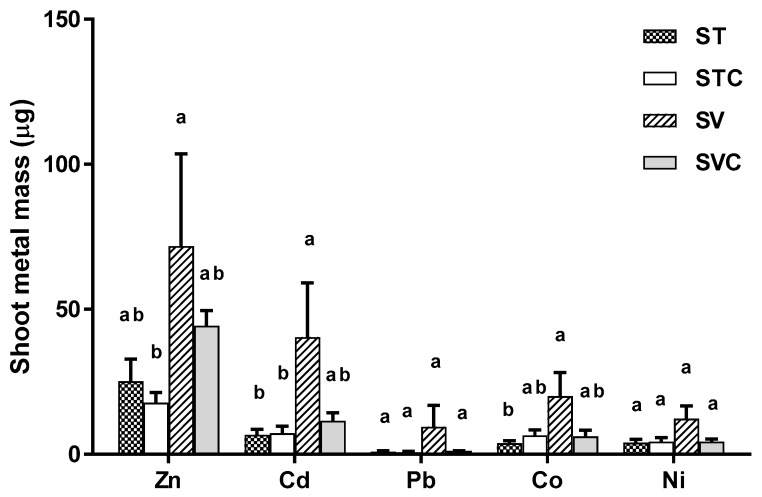
Mean ± 1 S.E. switchgrass shoot metal mass (μg) in soil (S) amended with thermophilic (T) and vermicompost (V) with and without coir (C). Varying letters indicate significant differences in shoot metal mass across treatments for each metal species.

Not enough switchgrass shoot and root biomass was recovered from the control treatment, while not enough root biomass was recovered from any of the treatments over the study duration for heavy metal determination. Nevertheless, shoot heavy metal mass was still determined in the organic treatments. SV treatments had the greatest shoot metal mass for all metal species, significantly differing from ST for Cd and Co (Figure 3). No significant differences in mass uptake were observed among the remaining treatments. Relative to the other trace metals (Cd, Pd, Co, and Ni), Zn uptake was the highest (2 to 13 times higher in mass) in each of the treatments (Figure 3). In all treatments, total soil metal mass at the end of the experiment was lower for all metals (Table 6) relative to their initial conditions (Table 3) in the vegetated control treatment except for Cd which increased slightly. On average, mass of Zn, Cd, and Pb was lower in soils with switchgrass than without, while the reverse was observed for Co and Ni.

### 3.5. Nutrient (PO_4_^3−^, NO_3_^−^, and NH_4_^+^) Leachate Concentrations

**Figure 4:**
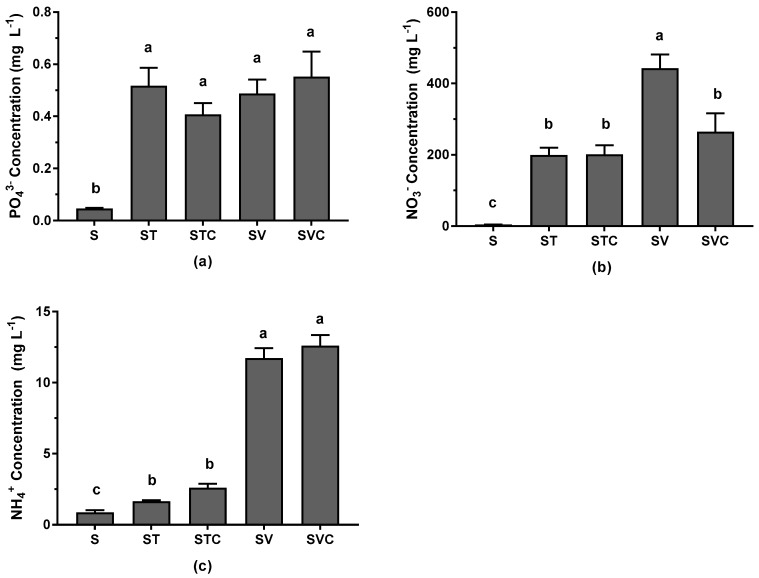
(**a**–**c**) Mean ± 1 S.E. PO_4_^3−^, NO_3_^−^, and NH_4_^+^ concentrations analyzed in the leachate from control soil (S), and control soil amended with thermophilic (T) and vermicompost (V) with and without coir (C) from pots containing no plants. Varying letters indicate significant differences in nutrient leachate concentrations across treatments.

Soils receiving organic amendments leached significantly higher nutrients than the control soil with no amendments (Figure 4). Between the two compost types, nutrient leachate was the highest from soils amended with vermicompost (SV and SVC). While there were no significant differences between the compost types for leachate PO_4_^3−^, leachate NO_3_^−^ concentrations were significantly higher from vermicompost without coir, and NH_4_^+^ concentrations in the leachate was significantly higher from vermicompost-amended soils with and without coir (Figure 4). Leachate NO_3_^−^ in S, ST, STC, SV, and SVC treatments were approximately 5, 122, 77, 38, and 21 times greater than leachate NH_4_^+^ s in the respective treatments.

## 4. Discussion

### 4.1. Effect of Organic Amendments on Metal Bioavailability

Composts addition to heavy metal contaminated soil significantly reduced bioavailable fraction of all metal constituents (Table 4). Soils naturally reduce solubility and mobility of heavy metals through sorption, precipitation, and complexation reactions [25,43]. Organic amendments to soils can accelerate this natural attenuation process [44] by increasing cation exchange capacity [20]—which increases soils’ ability to bind with heavy metals, rendering them less transportable—and microbial immobilization [45]. A study by O’Dell et al. [46] showed that addition of yard waste-derived compost rich in humic and fulvic acid favored the fixation of heavy metals in an acidic Cu–Zn minespoil, and reduced bioavailable concentrations of Cu and Zn. Complexes of some metals like Pb are found to be more stable (i.e., less bioavailable) than other metal complexes such as Cd (Table 4; [47]), which was also observed in the current study (Table 4). Soil pH also affects metal solubility. The control soil was more acidic with pH of 4.63 contrary to organically amended soils with pH of 6.42 to 6.79 (Table 5). Reduced pH can result in much higher metal solubility [48], which could explain why metal bioavailability was significantly higher in control soils. Chuan et al. [49] observed higher metal solubilities of Zn, Cd, and Pb under slightly acidic conditions (pH = 5). In contrast, increased pH due to composts can induce gradual alkalization of the soil, favoring the formation of metal hydroxides and carbonate complexes [25,50,51], which can decrease metal bioavailability.

### 4.2. Effects of Organic Amendments on Switchgrass Productivity and Soil Properties

Composts also improve soil properties. All the organic amendments containing compost alone, and compost plus coir, lowered soil acidity by increasing soil pH and EC (Table 5), as in other studies [26,36]. The pH observed in the amended treatments with plants ranged from 6.08 to 6.40 (Table 5), which is in the optimal range for switchgrass [52]. In contrast, soil pH in the control was outside the range considered suitable for switchgrass, which may have negatively affected plant growth (Table 5, Figure 2). By enhancing plant productivity on heavy metal-contaminated soil (Figure 2), the ability of plants to absorb (“phytoextract”) and bioaccumulate heavy metals from the soil [53] can be made possible as a long-term phytoremediation strategy. While organic amendments reduce solubility of heavy metals, they increase plant survival and productivity as shown by acting as slow-release fertilizers [54], which contributes to the long-term success of the phytoremediation strategy.

Switchgrass present in the organically amended soils had measurable levels of heavy metals in their shoots (Figure 3). Shoot concentrations of metals varied but were present in the order Zn > Cd > Co > Ni > Pb. Zn is a micronutrient essential for plant growth, so it is not surprising that they were present in the shoots in much higher concentrations compared to other metals. Other less essential metals for plant growth which can also be removed from soils via phytoextraction are Co, Ni, Fe, Mn, Cu, and Mo [55]. Plants are also successful in absorbing metals that lack a known biological function, such as Cd, Pb, and Cr [8,53,56]. Plant roots release organic compounds (e.g., chelators) which, along with plant-induced pH changes, enhance the solubility of adsorbed metals in the soil, and in turn, facilitate their uptake by plants even at low concentrations and from nearly insoluble precipitates [54]. If the growth had continued longer than the duration of our experiment, additional growth may have extracted metals from a greater soil volume through a more extensive root system [5]. In contrast to the amended soils, the control soil did not produce sufficient amounts of shoots or roots for analysis during the study period. This likely means that metal uptake is negligible when organic amendment was absent. Additionally, in the vegetated control treatments, toxicity from bioavailable metals may have harmed the roots due to lack of sorption mechanisms that keep metals insoluble [43,57].

The study shows that organic amendments boost plant survival and improve nutrient availability (Figure 2 and Figure 4) and soil properties (Table 5) on contaminated soils, while reducing metal bioavailability. In this experiment, plants assisted with heavy metal uptake, but over the time period we examined, it was not a major effect. In this study, there were no significant differences in plant production among the organic amendments (Figure 2), despite having large variations in inorganic N in soil and leachate. There were large differences in N between the two compost types (Table 2, Figure 4). The lack of difference in plant biomass between the two compost treatments could be attributed to plants being very young over the study duration and—due to initial fertilizer applications in the pots—*N* may not have been limiting. If the study duration had been extended, differences in plant biomass may have developed between the two compost treatments, due to large differences between their nutrient supplies (Table 2). Coir did not have significant effects on plant biomass as it contains few nutrients itself [31,35], but coir can improve soil performance overtime by increasing nutrient and water retention capacity [35]. It could be that the amount of coir utilized was limited to cause a statistical treatment effect. Increasing the ratio of coir in the mix may result in greater treatment effect of coir, but this needs to be investigated.

The decrease in the total metal mass from the pots at the end of the experiment (Table 6) can be attributed to uptake of metals by switchgrass. In unvegetated pots, metals may have leached out from the soil during the watering process carried out for the nutrient leachate experiment. Some portion of metals may also have leached out of the soils during the weekly watering process in all the pots. Though we tried to pour the leachate back into the pots, it may be possible that we were unable to re-capture all the metals in time, due to possible adsorption of metals to the plastic container [58,59]. Cd was the only metal found in slightly higher mass at the end of the study in two of the pots (Table 6). The discrepancy was small, and could result from detection limit of the instrument, as Cd was present in very low concentrations in all the initial substrates (Table 2). As expected, total metal mass was generally lower, but not always in the vegetated pots. Lack of noticeable differences may be due to small amounts of metal uptake by switchgrass over the study duration (Figure 3) compared to the large amount of metal that was added (Table 3). The watering process for leachate nutrient analysis was also only conducted in the unvegetated pots, thus loss of metals during these processes could have resulted in treatment differences that were smaller than expected.

### 4.3. Phytoremediation Trade-Offs with Water Quality

Use of compost for phytoremediation of post-industrial sites (contaminated with heavy metals) or in green stormwater infrastructure sites (e.g., bioretention) for stormwater treatment of heavy metals, may not always affect water quality positively. This is because nutrients can be leached from compost during and following wet events (e.g., rainfall, irrigation), which can pollute surface or groundwater. The resulting leachate nutrient concentrations from compost-amended soils were significantly greater than the control soil, even when compost made up as little as 8% of the total soil mix (Figure 4). The type of compost also controls the concentrations released in the leachate. We observed significantly higher NO_3_^−^ concentrations from SV treatments relative to SVC, ST, and STC treatments. This is most likely due to lower CN ratio (Table 2), and higher extractable NO_3_^−^ concentrations of vermicompost compared to compost (2230 vs. 505 mg L^−1^ respectively; Table 2). Hurley et al. [41] also observed significantly higher NO_3_^−^ concentrations in the leachate originating from vermicompost compared to leachate from four different composts samples. Frederickson et al. (2007) [60] observed a similar trend of significantly higher extractable NO_3_^−^ concentrations (2660 mg kg^−1^) from vermicompost relative to compost (1531 mg kg^−1^). The addition of coir to the compost-amended soils did not significantly influence nutrient release in the leachate, except for NO_3_^−^ which was significantly reduced in the SVC relative to SV (Figure 4). Coir, which provides an additional carbon source [36] in the SVC treatment, may have stimulated microbial biomass and activity leading to increased immobilization of NO_3_^−^ [61].

While there were no differences in leachate PO_4_^3−^ concentrations between the two compost types, NH_4_^+^ leachate concentrations were significantly greater from the vermicompost treatments with and without coir (Figure 4). This is attributed to the higher (34 times) extractable NH_4_^+^ concentrations in the original vermicompost sample relative to the compost (Table 2). Higher NH_4_^+^ concentrations also suggest the potential for high nitrification rates, which were indicative of the vermicompost treatments. Nitrates, the end products of nitrification reactions, are extremely mobile anions [62], and hence, leach out easily from the soil. This means that, depending on the compost type, an optimum proportion of compost and soil mix must be determined to ensure success for phytoremediation, while minimizing nutrient leaching potential. If compost with higher nutrient leaching potential is being applied to soils, appropriate best management practices should be implemented to minimize nutrient mobilization into sensitive water bodies.

## 5. Conclusions

Our research indicates that the effectiveness of phytoremediation can be increased by amending heavy metal-contaminated soil with composts (thermophilic or vermicompost). Addition of organic amendments reduced metal solubility, and increased soil pH, EC, and soil nutrient status. Organic amendments significantly improved switchgrass productivity compared to the non-amended control. Switchgrass in the amended treatments showed detectable levels of metal uptake in shoots, but extremely low growth in the non-amended soil suggests negligible metal uptake. However, if the study duration is extended, and switchgrass continues to accumulate more aboveground and belowground biomass, this will likely increase the total metal uptake potential of switchgrass, which could further prevent losses of bioavailable heavy metals, mineralized N (e.g., particularly NO_3_^−^ which is mobile), and PO_4_^3−^ to the leachate. On the other hand, metal-contaminated soils deprived of organic matter can increase metal bioavailability, subsequently increasing toxicity to plants. This hampers plant survival and performance (plants ability to uptake and sequester metals), thereby undermining phytoremediation as a pollutant control strategy.

Some confounding factors in the study that were not controlled for are the maturity/age and feedstocks used to create the two composts; however, this should not have interfered with the results observed. We believe that by having an additional treatment of soil and coir alone, it would be possible to detect the effects of coir alone. Due to water quality implications of compost arising from nutrient leaching, which can pollute surface and groundwaters, the amount of compost deemed necessary for phytoremediation must consider the effects on plant productivity and nutrient pollution in nutrient-sensitive watersheds. Applying compost at levels targeted to fulfill specific crop N and P demand can help minimize negative effects.

## Figures and Tables

**Table 1:** Experimental treatments.

Substrate Type	Composition	Plant	No Plant
Soil	Soil	S	S
Soil + Thermophilic Compost	92% Soil: 8% compost	ST	ST
Soil + Thermophilic Compost+ Coir	88% Soil: 8% compost + 4% coir	STC	STC
Soil + Vermicompost	92% Soil: 8% compost	SV	SV
Soil + Vermicompost + Coir	88% Soil: 8% compost: 4% coir	SVC	SVC

S: Soil, P: Plant, T: Thermophilic Compost, V: Vermicompost, C: Coir.

**Table 2:** Chemical properties of the soil, composts (thermophilic and vermicompost), and coir.

Parameters	Soil	Therm. Compost	Verm. Compost	Coir
Organic matter (%)	0.7	37.5	33.1	41
CN ratio	13.6	13.61	10.3	84
Total *N* (%)	0.2	1.54	1.8	0.5
NH_4_^+^, ppm		1.78	60.3	0.73
NO_3_^−^, ppm		505	2230	0.02
pH	4.9	8.09	7.1	5.6 *
Effective CEC, meq/100g	0.6			
Total heavy metals, ppm				
Copper (Cu)	18.2	50.9	841	12.7
Zinc (Zn)	68.1	147	660	12
Cadmium (Cd)	<0.2	<0.2	<0.2	<0.2
Chromium (Cr)	35.9	17.1	2.6	1.1
Lead (Pb)	16.9	32	9.2	1.2
Nickel (Ni)	27	13.8	7.8	2.6
Cobalt (Co)	9.2	4.8	1.2	0.2

* [31].

**Table 3:** Total mass (mg) of metals in soil per pot in each treatment after contamination of the soil.

	Total Zn	Total Cd	Total Pb	Total Ni	Total Co
	mg/pot				
S	148.01	33.94	61.99	78.96	49.06
ST	140.27	33.91	60.03	75.46	47.87
STC	135.72	33.90	58.90	73.66	47.25
SV	145.19	33.91	59.81	75.41	47.83
SVC	140.65	33.90	58.68	73.60	47.21

S = Soil, T = Thermophilic compost, C = Coir, V = Vermicompost.

**Table 4:** Bioavailable metal mass (mg) in soil per pot from control soil (S) and soils amended with thermophilic (T) and vermicompost (V) with and without coir (C) from unvegetated pots. Numbers inside parenthesis indicate ± 1 S.E. (*n* = number of replicates/pots).

Treatment	*n*	Zn	Cd	Pb	Co	Ni
		mg/pot				
S	3	20.104 ^a^ (0.837)	23.611 ^a^ (0.875)	0.204 ^a^ (0.010)	23.178 ^a^ (0.909)	25.225 ^a^ (0.632)
ST	4	0.342 ^b^ (0.058)	0.282 ^b^ (0.067)	0.027 ^b^ (0.016)	0.304 ^b^ (0.089)	0.388 ^b^ (0.089)
STC	4	0.231 ^b^ (0.089)	0.345 ^b^ (0.021)	0.030 ^b^ (0.021)	0.450 ^b^ (0.063)	0.456 ^b^ (0.030)
SV	4	0.442 ^b^ (0.115)	0.294 ^b^ (0.024)	0.042 ^b^ (0.022)	0.355 ^b^ (0.023)	0.529 ^b^ (0.046)
SVC	4	0.704 ^b^ (0.077)	0.309 ^b^ (0.048)	0.028 ^b^ (0.014)	0.449 ^b^ (0.073)	0.568 ^b^ (0.075)

^a,b^ Varying letters indicate significant differences across treatments for each metal species at *p* < 0.05.

**Table 5:** Soil pH and electrical conductivity (EC) (μS cm^−1^) from control soil (S), and control soil amended with thermophilic (T) and vermicompost (V) with and without coir (C) from unvegetated (−) and vegetated (+) pots. (*n* = number of replicates/pots).

Plants	Treatment	*n*	pH	EC (μS cm^−1^)
(−)	S	3	4.63 ^b^ (0.12)	84 ^b^ (15)
	ST	4	6.79 ^a^ (0.06)	364 ^a^ (49)
	STC	4	6.53 ^a^ (0.15)	324 ^a^ (26)
	SV	4	6.44 ^a^ (0.14)	917 ^a^ (51)
	SVC	4	6.59 ^a^ (0.04)	1219 ^a^ (104)
(+)	S	3	4.67 ^c^ (0.14)	81 ^c^ (0.99)
	ST	4	6.40 ^a^ (0.04)	255 ^b^ (27)
	STC	4	6.34 ^a^ (0.06)	234 ^b^ (8.3)
	SV	4	6.08 ^b^ (0.10)	812 ^a^ (99)
	SVC	4	6.29 ^b^ (0.03)	776 ^a^ (71)

^a,b,c^ Varying letters indicate significant differences across treatments at *p* < 0.05.

**Table 6:** Total heavy metal mass in control soil (S), and control soil amended with thermophilic (T) and vermicompost (V) with and without coir (C) from unvegetated (−) and vegetated (+) pots at the end of the 54-day incubation period. Numbers inside parenthesis indicate ± 1 S.E. (*n* = number of replicates/pots).

Plants	Treatment	*n*	Zn	Cd	Pb	Co	Ni
			mg				
(−)	S	3	105.38 ^b^ (9.65)	33.09 ^a^ (0.60)	47.52 ^a^ (3.63)	38.54 ^a^ (2.55)	57.54 ^a^ (3.53)
	ST	4	119.24 ^a,b^ (3.63)	35.03 ^a,b^ (0.97)	55.49 ^a^ (1.52)	33.67 ^a,b^ (0.79)	46.51 ^b^ (0.24)
	STC	4	110.63 ^b^ (6.72)	29.87 ^b^ (1.26)	48.32 ^a^ (3.16)	29.36 ^b^ (1.16)	42.77 ^b^ (1.65)
	SV	3	143.44 ^a^ (4.89)	32.81 ^a,b^ (2.01)	50.56 ^a^ (4.24)	32.13 ^b^ (0.89)	42.96 ^b^ (1.90)
	SVC	4	136.88 ^a^ (4.70)	30.93 ^b^ (2.45)	46.63 ^a^ (3.77)	30.53 ^b^ (0.98)	43.10 ^b^ (1.26)
(+)	S	3	102.69 ^a^ (3.02)	34.68 ^a^ (1.28)	50.60 ^a^ (1.85)	40.12 ^a^ (1.22)	55.52 ^a,b^ (1.43)
	ST	4	110.45 ^a^ (4.11)	31.25 ^a,b^ (1.73)	51.76 ^a^ (1.89)	33.55 (1.02)	58.80 ^a^ (8.42)
	STC	4	109.91 ^a^ (5.00)	29.32 ^b^ (3.71)	48.86 ^a^ (4.43)	40.75 ^a^ (8.26)	47.57 ^a,b^ (2.72)
	SV	4	120.28 ^a^ (2.72)	25.42 ^b^ (0.29)	44.57 ^a^ (1.58)	28.93 ^a^ (0.84)	42.29 ^a,b^ (0.76)
	SVC	4	117.31 ^a^ (4.45)	24.18 ^b^ (1.14)	43.19 ^a^ (0.86)	26.09 ^a^ (0.57)	39.71 ^b^ (1.35)

^a,b,c^ Varying letters indicate significant differences across treatments for each metal species within vegetated and unvegetated pots at *p* < 0.05.

## Appendix A

**Table A1:** Total mass of metal in each substrate used in the experiment and calculated total mass of metals in the different treatments before contamination.

**Substrate**	**Weight (kg)**	**Total Mass (mg) of Metal in Each Experimental Substrate**				
		**Zn**	**Cd**	**Pb**	**Ni**	**Co**
Soil	1.6	114.408	0.336	28.392	45.36	15.456
Therm. Compost	0.12	17.64	0.024	3.84	1.656	0.576
Vermicompost	0.12	79.2	0.024	1.104	0.936	0.144
Coir	0.06	0.762	0.012	0.072	0.156	0.012
	**Treatment**	**Total Mass (mg) of Metal in Each Treatment before Contamination**				
		**Zn**	**Cd**	**Pb**	**Ni**	**Co**
	S	114.41	0.34	28.39	45.36	15.46
	ST	106.67	0.31	26.43	41.86	14.27
	STC	102.12	0.30	25.30	40.06	13.65
	SV	111.59	0.31	26.21	41.81	14.23
	SVC	107.05	0.30	25.08	40.00	13.61
